# Quantification and Predictors of Hemoglobin Drop, Hidden Blood Loss and Irrigation Fluid Retention in Shoulder Arthroscopy

**DOI:** 10.3390/jcm14113875

**Published:** 2025-05-30

**Authors:** Nikola Matejcic, Nikola Grzalja, Karlo Tudor, Andrica Lekic, Filip Stefanac, Ana Matejcic, Lana Ruzic

**Affiliations:** 1 Lovran Orthopaedic Clinic, Faculty of Medicine, University of Rijeka, 51415 Lovran, Croatia; 2 Faculty of Health Studies, University of Rijeka, 51000 Rijeka, Croatia; 3 Independent Researcher, 51000 Rijeka, Croatia; 4 Faculty of Kinesiology, University of Zagreb, 10000 Zagreb, Croatia

**Keywords:** arthroscopy, blood loss, surgical, hematocrit, hemodilution, hemoglobin, low-molecular-weight heparin, shoulder

## Abstract

**Background**: Shoulder arthroscopy is a common, minimally invasive surgery, but the resulting postoperative blood loss remains poorly understood. In this study, we quantified the intraoperative and postoperative blood loss, the hemoglobin (Hb) drop, and the effects of irrigation fluid retention, as well as the influence of solutions administered through infusions. **Methods**: A prospective observational study of 49 patients undergoing arthroscopic rotator cuff tear (RCT) repair was conducted. Their preoperative and postoperative Hb levels were measured, along with the intraoperative and postoperative blood loss. Irrigation fluid retention was analyzed, and multiple regression was used to assess the factors contributing to Hb drops. **Results**: The intraoperative blood loss amounted to 36.46 ± 20.34 mL, while the total blood loss reached 791.17 ± 280.96 mL, with 94.64% occurring postoperatively. The postoperative Hb drop (2.06 ± 0.74 g/dL) was significantly greater than the intraoperative Hb drop (0.11 ± 0.06 g/dL) (*p* < 0.001). An older age (*p* = 0.02) and male sex (*p* = 0.025) significantly predicted the postoperative Hb drop, while irrigation fluid retention and administration of crystalloids and colloids had no notable effects. Capsulotomy was associated with a small but significant increase in intraoperative blood loss (*p* < 0.01). Increased intraoperative blood loss correlated with greater irrigation fluid retention (r = 0.41, adjusted R2 = 0.152, *p* < 0.001). **Conclusions**: In shoulder arthroscopy, the postoperative blood loss and Hb drop are significantly greater than the intraoperative blood loss and Hb drop, as well as the fluid gain, emphasizing the need for careful monitoring, especially in high-risk patients. Future studies should investigate the potential impacts of low-molecular-weight heparin on postoperative bleeding after shoulder arthroscopy.

## 1. Introduction

Shoulder arthroscopy is a minimally invasive surgical method that has evolved significantly in recent decades, leading to the expansion of its use in the treatment and diagnosis of shoulder girdle diseases and injuries [1,2]. Given its advantages, alongside the well-known benefits of minimally invasive techniques compared to traditional surgical methods, there has been a notable shift towards increased utilization, and it is often performed as an ambulatory procedure [3,4,5]. The growing demand for shoulder arthroscopic procedures has heightened the awareness of the need for safe and efficient surgical practices that achieve optimal clinical outcomes while minimizing complications. Arthroscopic rotator cuff tear (RCT) repair has been gradually established as one of these procedures [6,7,8]. A fundamental requirement for safe surgery, precision, and shorter operations is high-quality visualization, which is reflected in the visual clarity on the lens of the arthroscope. Visual clarity directly depends on the amount of blood in the irrigation solution used during the procedure, making bleeding control crucial [9]. This is achieved through various measures, including hypotensive anesthesia, regulating the pressure of the irrigation fluid (typically maintained using arthroscopic pumps), turbulence control, hemostasis via radiofrequency ablation, the use of epinephrine (EPI) in the irrigation fluid, or the administration of tranexamic acid (TXA) intravenously or intra-articularly [10]. Controlled hypotensive anesthesia allows for reduced intraoperative blood loss, a lower pressure requirement for the irrigation fluid, and shorter operations, thus minimizing the risk of harmful fluid extravasation into the surrounding soft tissues. During the procedure, the mean arterial pressure (MAP) should not drop more than 20–30% below the patient’s usual MAP in order to prevent ischemic complications, and the baseline irrigation fluid pressure is typically around 50 mmHg [11,12,13]. The interest in blood loss during shoulder arthroscopy is primarily related to intraoperative loss, as it directly affects visibility. To date, three studies have objectively and accurately measured the hemoglobin (Hb) concentration in waste irrigation fluid as an indicator of intraoperative bleeding during shoulder arthroscopy. Jensen et al. demonstrated the positive effects of EPI on visual clarity and bleeding in experimental and control groups undergoing diagnostic and therapeutic arthroscopies [9]. Griffiths et al. calculated the intraoperative blood loss and irrigation fluid retention during various shoulder arthroscopies, noting a clinically insignificant Hb drop. These findings support the claim that shoulder arthroscopy is a safe day-case procedure, although the authors did not measure its effect on visibility [14]. Liu et al. measured the Hb concentration in the waste irrigation fluid during arthroscopic RCT repair, although they did not calculate the intraoperative blood loss. Another study showed unexpectedly high total perioperative blood loss and proved the positive effect of TXA on visual clarity [15]. The total blood loss is more challenging to objectively evaluate due to the absorption of retained irrigation fluid from the surrounding shoulder tissues into the systemic circulation, leading to hemodilution and Hb drift [13,16,17,18]. The dynamics of irrigation fluid absorption remain poorly understood and are influenced by various factors. For reference, the resorption rate of saline during subcutaneous infusions in palliative care for elderly multimorbid patients is approximately 127 mL/h [19]. Several studies have analyzed the total blood loss and Hb drop during arthroscopic RCT repair. Guo et al. and Lee et al. analyzed the risk factors for increased blood loss, with both studies involving the use of EPI in the irrigation fluid [20,21]. The clinical study by Apivatgaroon and Ratanacharatroj analyzed the efficacy of postoperative drainage after arthroscopic RCT repair [22]. Other clinical studies have compared the effects of TXA in experimental and control groups on the visual clarity, blood loss, and operating time (Kawaguchi et al.); its effects on blood loss, the postoperative inflammatory response, and pain (Wang et al.); and its impacts on the total blood loss and postoperative pain in the context of the use of EPI in the irrigation fluid (Zhu et al.) [23,24,25]. These studies all reported significantly greater total blood loss and Hb drops than the previously mentioned studies that measured the intraoperative loss, indicating greater postoperative bleeding and the presence of “hidden blood loss”, as well as possible Hb drift. An Hb drop greater than 1 g/dL can be taken as clinically significant [14]. Periarticular bleeding and hemarthrosis are clinically important due to their potential to increase the risk of infectious complications, cartilage damage, synovitis, increased pain, swelling, and delayed rehabilitation [26]. Several studies have also analyzed irrigation fluid retention and its impact on blood physiology, the cardiovascular system, respiratory compromise, measurable anthropometric parameters, and clinical outcomes [16,17,27,28]. Due to their interrelation, we believe that the analysis of Hb drops and bleeding—both intraoperative and postoperative—and the total blood loss cannot be entirely separated from the analysis of irrigation fluid retention and fluid intake through infusions, especially colloids, as this relationship is not yet fully understood.

The main objective of the present study was to determine the extent of the Hb drop caused by immediate intraoperative bleeding and calculate the total Hb drop and blood loss; based on this, we sought to assess the postoperative Hb drop and blood loss. Moreover, we aimed to examine the impacts of demographic, intraoperative, and postoperative variables on intraoperative and postoperative bleeding and Hb drops.

In particular, we hypothesized that, in the context of shoulder arthroscopy, the intraoperative Hb drop and blood loss would be statistically significantly lower than the postoperative Hb drop and blood loss. The second hypothesis was that the volume of retained irrigation fluid and the volumes of crystalloids and colloids administered via infusion would affect the postoperative Hb drop.

## 2. Materials and Methods

### 2.1. Patients

An observational study was conducted at the Lovran Orthopedic Clinic of the Faculty of Medicine of the University of Rijeka, Croatia, from June 2021 to July 2022. It included 49 patients undergoing arthroscopic RCT repair. The inclusion criteria were an age >18 years and a diagnosis of RCT that was previously confirmed via both ultrasound and magnetic resonance imaging. Patients with a history of coagulopathy, an abnormal coagulation profile, renal or liver disorders, and uncontrolled hypertension (systolic blood pressure ˃ 180 mmHg), and those who were receiving anticoagulant and antiplatelet medication before surgery, were excluded.

In our study, we analyzed the Hb drop, bleeding, and irrigation fluid retention in the same cohort of patients undergoing arthroscopic RCT repair and related procedures. We examined their causes and mutual influences. For postoperative blood sampling and analysis, we chose the second postoperative day as the day that was most likely to yield the lowest Hb concentration, or the beginning of the period during which the lowest Hb concentration was expected following blood loss [29,30,31]. We aimed to investigate the worst-case scenario. This time point was also presumed to correspond to the near-complete resorption of retained irrigation fluid and the clearance of any administered colloids [19,32].

This study was conducted in accordance with the Declaration of Helsinki, and the protocol was approved by the Ethics Committee of the Lovran Orthopedic Clinic of the Faculty of Medicine of the University of Rijeka, Croatia (protocol code: 02-341/2021, date of approval: 11 May 2021). Informed consent was obtained from all patients involved in the study.

### 2.2. Surgical Technique and Early Postoperative Care

All patients underwent surgery in the beach chair position. It was performed by a single experienced surgeon and a hospital resident under general hypotensive anesthesia, combined with an ultrasound-guided interscalene brachial plexus block administered by a team of anesthesiologists. Before the start of the procedure, mechanical thromboprophylaxis measures were applied through the use of elastic stockings. The blood pressure was measured every 5 min on the contralateral arm using an automatic blood pressure cuff, which was controlled and adjusted as necessary by the anesthesiologists. Crystalloid (saline and Ringer’s solution) and colloid (6% HES 130/0.4) solutions were administered intravenously at the discretion of the anesthesiologists. During the procedure, a 4 mm/30° arthroscopic lens was used, and the irrigation fluid was normal saline without EPI or TXA, maintained at a pressure of 50 mmHg via an arthroscopic pump, with the option of a temporary pressure increase of 20 mmHg for 2 min in cases of bleeding. Hemostasis was achieved using a radiofrequency device. The arm was placed under 2.5 kg of traction. Cannulas were not used. The primary surgical procedure was arthroscopic RCT repair. Supraspinatus (SSP) tendon lesions, whether isolated or in combination with infraspinatus (ISP) tendon lesions as part of a posterosuperior tear, were repaired using a single- or double-row technique, depending on the size and tension of the tear, with tendon cleavage alignment and margin convergence sutures if needed. The single-row technique applied was a complex type using modified Mason-Allen sutures and triple-loaded suture anchors. The double-row technique was performed using one or two anchors in both the medial and lateral rows, employing double- or triple-loaded suture anchors and knotless anchors [33,34,35].

Subscapularis (SSC) tendon tears were repaired using 1–2 suture anchors, based on the size of the tear [36]. If necessary, one or more of the following additional surgical procedures were performed: long head of the biceps brachii (LHBB) tenodesis or tenotomy, glenohumeral (GH) stabilization-capsular shift, labral repair, acromioclavicular (AC) joint resection, acromioplasty, subacromial bursectomy and soft tissue release (SA-BSTR), subcoracoid bursectomy and soft tissue release (SC-BSTR), synovectomy, capsulotomy, microfractures (MFX), tuberoplasty, biopsy, and Ca deposit evacuation [37,38]. LHBB tenodesis, GH stabilization, and labral repair were performed using all-suture anchors. The implants used included 5.5 mm titanium suture anchors (Corkscrew FTII and FTIII; Arthrex Inc., Naples, FL, USA), 5.5 mm knotless biocomposite anchors (SwiveLock; Arthrex Inc., Naples, FL, USA), and 1.5 mm and 2.9 mm all-suture anchors (JuggerKnot; Biomet Sports Medicine, Warsaw, IN, USA). At the end of the procedure, hemostasis was checked, and it was additionally performed if necessary. Drains were not used [22]. Postoperatively, the arm was immobilized in a sling, and thromboprophylaxis with low-molecular-weight heparin (nadroparin calcium 3800 IU subcutaneously) was initiated in accordance with the hospital protocol [39,40,41]. Rehabilitation, supervised by a physiotherapist, began on the first postoperative day, continuing until the patient achieved full independence. Patients were typically discharged on the second postoperative day.

### 2.3. Data Collection

On the day of hospital admission, at least 24 h before surgery and prior to fasting, each patient’s weight, height, and body mass index (BMI) were measured, and blood samples were taken for Hb, hematocrit (Hct), red blood cell (RBC), white blood cell (WBC), and platelet (PLT) counts. The coagulation profiles were also assessed.

During surgery, data were collected on the number of torn tendons, the size of SSP and ISP lesions in mm, the condition of the SSC tendon, and the presence of synovitis. The duration of the procedure, the mean arterial pressure (MAP), the number of times that the pump pressure was increased by 20 mmHg, and the irrigation fluid inflow and outflow volumes were recorded. The procedure was thoroughly documented, including all the surgical steps performed and the number of implants used.

All perioperative intravenous infusions (crystalloid and colloid solutions) were recorded. Waste irrigation fluid was collected; it was homogenized via careful mixing and prepared in the hospital laboratory, and a sample was sent to the Department of Medical Chemistry, Biochemistry, and Clinical Chemistry, Faculty of Medicine, University of Rijeka, Croatia, where the Hb concentration in each sample was determined spectrophotometrically (Varian Cary 100 Bio, 190–900 nm, resolution ≤ 0.189 nm, wavelength accuracy ±0.02 nm to ±0.04 nm). The described protocol represents a modification of the Cripps method, which is detailed in the article by Griffiths et al. [14,42].

Using the equations described by Griffiths et al., the intraoperative blood loss (in mL) and Hb drop (in g/dL) were calculated [14]. Surgical drapes were weighed before and after surgery, and the weight difference was converted to a volume (1 L saline = 1009 g). The retained irrigation fluid volume was calculated using the following equation:

Retained Irrigation Fluid Volume (mL) = Inflow Volume − (Outflow Volume + Fluid from Surgical Drape Volume).

On the second postoperative day, blood samples were taken for the analysis of the same parameters as assessed preoperatively. The average postoperative MAP and potential complications were also recorded.

The total blood loss was calculated using the Gross formula based on the preoperative and postoperative Hct and the estimated blood volume determined via Nadler’s formula [43,44].

The postoperative Hb drop was determined using the following equation:

Postoperative Hb Drop (g/dL) = Total Hb Drop − Intraoperative Hb Drop.

The postoperative (hidden) blood loss was determined using the following equation:

Postoperative (Hidden) Blood Loss Volume (mL) = Total Blood Loss − Intraoperative Blood Loss.

### 2.4. Statistical Analysis

In this study, Statistica version 14.1.0.8 (Cloud Software Inc., Fort Lauderdale, FL, USA) was used. Statistical significance was assessed at *p* ≤ 0.05, corresponding to 95% confidence intervals. To achieve 80% power with r = 0.40, α = 0.05 (Type I error), and β = 0.20 (Type II error), it was estimated that a minimum of 46 subjects was needed. Descriptive statistics were used for data presentation, including the calculation of mean, standard deviation, minimum, and maximum (range) values. For nominal data, frequencies and percentages were used. The Kolmogorov–Smirnov test was applied to assess normality. Since the data followed a normal distribution, parametric tests were used, namely Student’s *t*-test for dependent samples. Correlations were expressed using Pearson’s correlation coefficient (r). Multiple regression analysis was used to identify potential predictors of the Hb drop. The figures were created using Microsoft Office Excel.

## 3. Results

In our cohort of 49 patients (22 females and 27 males), 21 (43%) presented with an isolated SSP tear, one patient (2%) had an isolated SSC tear, and one patient (2%) had an isolated ISP tear. Additionally, 18 patients (37%) exhibited combined SSC+SSP tears, four patients (8%) had SSP+ISP tears, and four patients (8%) presented with massive rotator cuff tears involving the SSC+SSP+ISP tendons. We found an SSC tear in 47% of our patients. All tendons were completely repaired. We used the double-row technique for 28.57% of the isolated SSP tears, 50% of the SSP+ISP tears, and 5.55% of the SSP combined with SSC tears, while 39% of the SSC tears were classified as Lafosse grade 3 or 4 and were repaired using two suture anchors. During the procedure, 57% of the patients received 0.5 L of a colloid solution via infusion. Detailed demographic data, as well as the intraoperative findings, fluid usage, Hb concentrations in washed irrigation fluid, and MAP, are presented in Table 1.

Hemodilution was assessed by comparing the preoperative and postoperative RBC, WBC, PLT, Hb, and Hct values, as shown in Table 2 [45]. A paired Student’s *t*-test revealed significant differences in the preoperative and postoperative Hb, Hct, RBC, and PLT values (*p* < 0.001), as well as the WBC value (*p* = 0.026). These results suggested the possibility of hemodilution.

To test hypothesis 2, we conducted a multiple regression analysis. We used age, sex, BMI, the operating time (minutes), the number of irrigation fluid pressure increases, the inflow irrigation fluid volume (L), irrigation fluid retention (L), crystalloids (L), and colloids (L) as predictor variables, while the postoperative Hb drop was the criterion variable. This analysis yielded a multiple regression coefficient of R = 0.567, which was statistically significant (*p* = 0.048). However, among the listed parameters, only an older age (*p* = 0.02) and male sex (*p* = 0.025) had a significant effect on the postoperative Hb drop.

The quantified Hb drop and blood loss parameters are presented in Table 3.

The Student’s *t*-test for dependent samples showed that the postoperative blood loss and Hb drop were significantly greater than their intraoperative values (*p* < 0.001).

A moderately significant correlation was observed between the patient’s age and the percentage of total blood loss (r = 0.329*), postoperative (hidden) blood loss (r = 0.319*), and postoperative (hidden) hemoglobin drop (r = 0.380*), with the significance level for all correlations set at *p* < 0.05.

No significant correlation was found between the blood loss parameters and MAP.

Intraoperative blood loss strongly predicted irrigation fluid retention, likely because increased blood loss necessitated higher irrigation fluid inflow. The linear regression analysis yielded a correlation coefficient of r = 0.41, an adjusted R^2^ of 0.152, F (1, 47) = 9.65, and *p* < 0.001.

The number of anchors used and the anchor rows in SSP/ISP repair did not exhibit an effect on any of the blood loss parameters.

**Figure 1:**
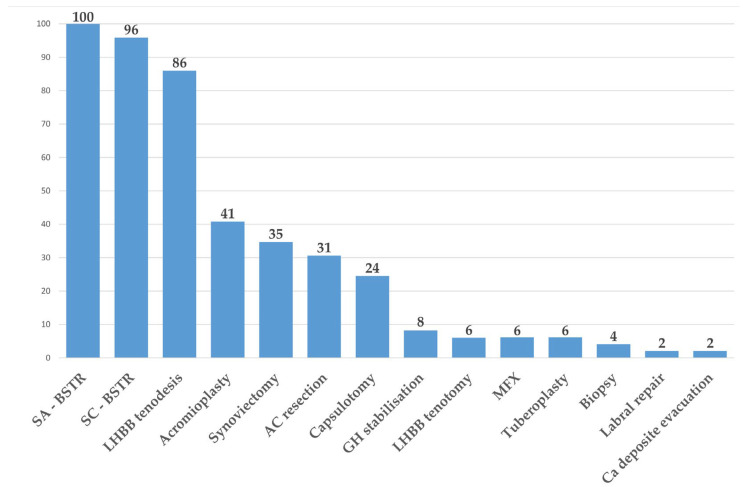
Additional procedures (% of patients). Abbreviations: SA-BSTR = subacromial bursectomy and soft tissue release; SC-BSTR = subcoracoid bursectomy and soft tissue release; LHBB = long head of the biceps brachii tendon; AC = acromioclavicular joint; GH = glenohumeral joint; MFX = microfractures.

During arthroscopy, several additional procedures were performed; thus, we assessed the potential influence of various procedures on the blood loss parameters. On average, the patients underwent 4.47 ± 1.02 additional procedures, with the number of procedures ranging from two to seven per patient. The percentage of patients that underwent each additional procedure is illustrated in Figure 1.

For each procedure, the sample was divided into two groups, and the differences were evaluated using Student’s *t*-test. This analysis was feasible only for additional procedures with two clearly defined groups, such as acromioplasty, AC resection, capsulotomy, and synovectomy. Since acromioplasty was performed as an additional procedure in 41% of the patients, we controlled for its potential influence on the blood loss parameters; however, no significant differences were observed. Similarly, AC resection and synovectomy did not significantly affect blood loss. Conversely, capsulotomy was associated with a statistically significant increase in intraoperative blood loss (*p* < 0.01), although this difference was likely clinically negligible, with an average increase of only 17 mL and high variability among the patients.

All patients underwent at least two different procedures. A multivariate analysis was performed to evaluate the effects of the additional procedures; however, no single procedure emerged as a significant predictor of blood loss. The multiple regression results were as follows: R = 0.464, R^2^ = 0.216, F (12, 36) = 0.827, *p* = 0.623, standard error of the estimate = 35.225. All beta coefficient values ranged from −0.24 to 0.30, with none reaching statistical significance, which was set at *p* < 0.05. Interestingly, the number of additional procedures was also not correlated with either the total blood loss (Pearson’s r = 0.11; *p* = 0.440) or the Hb drop expressed in g/dL (Pearson’s r = 0.09; *p* = 0.521).

During the hospital stay and follow-up after discharge, we did not observe any complications related to perioperative blood loss or fluid retention.

## 4. Discussion

Among the previous studies that have measured intraoperative bleeding through the precise determination of the Hb concentration in the irrigation fluid, our results can only be directly compared with those of Griffiths et al. [14].

In their study, Griffiths et al. measured the Hb concentrations in the irrigation fluid obtained from various well-defined therapeutic shoulder arthroscopies—25% intra-articular and 75% extra-articular procedures—of which 60% were subacromial decompressions and 40% were RCT repairs. The mean blood loss was 14.16 mL (range 1.22–36.45 mL), with significantly greater blood loss observed in extra-articular procedures. When calculated per unit of time, patients lost 0.26 mL/min, while this value was 0.33 mL/min when analyzing only extra-articular procedures. The intraoperative Hb drop was 0.035 g/dL, leading to the conclusion that the blood loss was clinically insignificant and that shoulder arthroscopy is suitable as a day-case procedure.

Our study is fully comparable with the above work, finding a mean intraoperative blood loss value of 0.29 mL/min and a greater total intraoperative blood loss value of 36.46 mL, along with an Hb drop of 0.11 g/dL. This was expected considering that all our procedures were extra-articular arthroscopic RCT repairs with multiple additional procedures.

The possibility of a comparison with the remaining two related studies is limited.

Jensen et al. analyzed a control group of 26 patients undergoing diagnostic and therapeutic shoulder arthroscopies and reported intraoperative blood loss of 12.87 ± 18.10 mL (range 0 to 87 mL) [9]. Since they did not specify the types of therapeutic arthroscopies performed, the ratio of diagnostic to therapeutic procedures, or the operating time, only an approximate comparison with the above-mentioned study can be performed.

Liu et al. performed a clinical study with a control group consisting exclusively of arthroscopic RCT repairs. They recorded an Hb concentration of 19.8 ± 40.3 µM without calculating the total intraoperative blood loss [15]. However, when the values were converted from µM to mg/mL, the Hb concentration in the irrigation fluid—and thus the intraoperative blood loss—in their study appeared to be significantly higher.

Nonetheless, a common finding across all studies was that the intraoperative blood loss and Hb drop were minimal and clinically insignificant, with relevance primarily in terms of visual clarity during arthroscopy [9].

On the other hand, the total blood loss and Hb drop during arthroscopic RCT repairs were surprisingly high in all studies, even in those that used EPI in the irrigation fluid.

In other studies, the total blood loss ranged from 322 to 542.5 mL, and the Hb drop ranged from 0.6 to 1.5 g/dL [15,20,21,22,23,25]. Wang et al. reported an intraoperative blood loss value of 106 mL in the control group, although the method of blood loss estimation was not specified [24].

A common feature among all the studies mentioned is that none reported the use of pharmacological thromboprophylaxis during the perioperative period.

In our study, the average total blood loss was calculated to be 791.17 mL, with an Hb drop of 2.06 g/dL, based on blood samples taken on the second postoperative day. We did not use either EPI or TXA in the irrigation solution. In accordance with the established hospital protocol, all of our patients received pharmacological thromboprophylaxis with low-molecular-weight heparin (nadroparin calcium 3800 IU subcutaneously once daily) after surgery, and, by the time of the second postoperative blood sampling, each patient had received two doses of the drug.

In our study, irrigation fluid retention amounted to 1.39 L/h, with a retention rate of 8.17%. Intraoperative blood loss emerged as a strong predictor of retention (*p* < 0.001). We did not use cannulas. Other studies have reported fluid retention ranging from 0.014 to 1.88 L/h, with retention rates between 0.2% and 29% [14,16,17,27,28,46,47]. The lowest retention was reported in the study by Syed et al., at 0.014–0.54 L/h, with a retention rate of 0.2–2.2%. In their study, they analyzed the use of cannulas and achieved better results with a fenestrated outflow cannula compared to a standard cannula without outflow [46].

Based on the measured total and intraoperative blood loss values, we calculated the amount of postoperative (hidden) blood loss, which averaged 754.72 mL, with an Hb drop of 1.95 g/dL. This accounted for 94.74% of the total blood loss and 94.01% of the total Hb drop. The difference between the intraoperative and postoperative (hidden) blood loss and Hb drops was statistically significant, thus confirming our first hypothesis (*p* < 0.001).

Bleeding associated with shoulder arthroscopy occurs predominantly after the surgical procedure is completed. Intraoperative bleeding is controlled by maintaining a lower MAP and regulating the pressure of the irrigation fluid using an arthroscopic pump [12]. The pressure of the irrigation fluid tamponades the capillary blood vessels, and additional hemostasis is achieved using a radiofrequency ablator. Once the procedure is completed, the MAP increases, the pump is turned off, the tamponade effect disappears, and bleeding is reactivated—resulting in what appears to be surprisingly large and hidden blood loss.

However, additional but difficult-to-measure factors include possible hemodilution and Hb drift, which may cause the blood loss and Hb drop to appear falsely elevated.

The total blood loss is calculated using the standard Gross formula, which adjusts for hemodilution and incorporates the pre- and post-blood-loss values of Hct or Hb [43]. After blood loss, the lowest Hb level is typically expected to occur between the second and fifth postoperative days [29]. In addition to actual blood loss, hemodilution takes place. This is partly influenced by fluid gain—referring to the volume of intravenous fluids given to the patient, particularly colloid solutions, which tend to remain in the bloodstream for a longer period. In shoulder arthroscopy, it also depends on the retention of the irrigation fluid, for which the rate of resorption into the bloodstream is unknown. The exact dynamics of these processes and the point at which the Hb concentration stabilizes after shoulder arthroscopy remain to be determined.

Other studies that measured the total blood loss collected blood samples on the first, third, and seventh days after surgery. In most studies, blood was drawn on the first postoperative day [15,22,25]. However, two studies took blood samples on both the first and seventh postoperative days, and, instead of observing recovery, they unexpectedly recorded a further Hb drop and greater blood loss on day 7 compared to day 1 [21,23]. In the study by Guo et al., the authors collected blood on the first and third days and also noted continued blood loss and Hb drops on the third day compared to the first postoperative day [20].

Taking into account these findings, as well as data from several studies indicating that the lowest Hb level may be expected on the second postoperative day, we chose to take blood samples on this day in order to theoretically examine the lowest Hb level [29,31]. Additionally, 57% of our patients received 0.5 L of a colloid solution via infusion during surgery. This further complicates the situation and can contribute to hemodilution—although 6% HES 130/0.4 should be cleared from circulation by the second day post-infusion [32].

We attempted to assess hemodilution by comparing the preoperative and postoperative Hb, Hct, RBC, WBC, and PLT values, and the findings of our statistical analysis indicated its possibility. Further statistical analysis was conducted to explore the potential influences of demographic characteristics, intraoperative variables, and factors that may affect fluid retention and hemodilution on the Hb drop. However, we were only able to confirm that an older age (*p* = 0.02) and male sex (*p* = 0.025) had a statistically significant effect on the postoperative Hb drop. Therefore, our second hypothesis—that the Hb drop would be caused by variables such as the volume of retained irrigation fluid and the volume of crystalloids and colloids administered via infusion, which could be associated with hemodilution—could not be confirmed.

An increased tendency towards bleeding in older patients was also reported in the study by Lee et al. [21].

Given the large number of additional procedures per patient (4.47 ± 1.02), we analyzed the potential individual and cumulative impacts of these procedures on blood loss and Hb drops. However, we found no statistically significant association between any single procedure or the total number of additional procedures and increased bleeding or Hb drops using multivariate methods. When analyzing the differences for each procedure individually, i.e., in cases where patients could be divided into those who had undergone the procedure and those who had not, only a capsulotomy was found to significantly increase the intraoperative blood loss (*p* < 0.01) and thus potentially affect visibility. The study by Guo et al. reported that capsulotomy might be a risk factor for total blood loss [20].

The administration of low-molecular-weight heparin in our study may also have contributed to increased postoperative bleeding, which should be explored in further analyses. The formation of postoperative wound hematomas is among the most common complications associated with its use [48].

Despite the potentially clinically significant estimated blood loss and Hb drops, we did not observe any clear clinical symptoms of bleeding, and the early postoperative course was uneventful; for example, all patients were able to participate in early rehabilitation.

Large perioperative blood loss in shoulder arthroscopy is not unusual. Studies that have analyzed bleeding in other endoscopic musculoskeletal procedures have also reported surprisingly high levels of hidden blood loss [45,49,50].

Key Strengths of the Study: This study was the first to precisely measure both intraoperative and postoperative contributions to the total Hb drop and blood loss in shoulder arthroscopy. In addition, the simultaneous measurement of irrigation fluid retention and the assessment of its impact on the Hb drop make this study unique.

Study Limitations: The dynamics and underlying processes of hemodilution and Hb drift after shoulder arthroscopy remain unclear. We believe that a more complete picture could be obtained by taking daily blood samples during the first seven postoperative days. As we were unable to confirm our findings statistically due to the potential influences of hemodilution and Hb drift, the total and postoperative blood loss values should be considered estimates. A more precise measurement of the retained irrigation fluid could have been achieved by weighing the patients immediately before and after surgery. Furthermore, all our patients received low-molecular-weight heparin during hospitalization. Future studies should investigate its potential impact on postoperative bleeding after shoulder arthroscopy.

## 5. Conclusions

Our study confirmed the existence of a statistically significant postoperative Hb drop and hidden blood loss compared to intraoperative levels in shoulder arthroscopy. These findings highlight the importance of postoperative monitoring and tailored management strategies for high-risk patients. Predictive factors for postoperative Hb drop and blood loss include older age and male sex, while intraoperative bleeding was identified as a predictive factor for irrigation fluid retention. Capsulotomy may increase the amount of intraoperatively lost blood. Future studies should further investigate fluid retention, the effects of hemodilution, and the potential role of low-molecular-weight heparin in postoperative bleeding after shoulder arthroscopy. Until these aspects are fully understood, we recommend an individualized approach to the administration of pharmacological thromboprophylaxis.

## Figures and Tables

**Table 1:** Descriptive data.

Variable	Mean	Std. Dev.	Minimum	Maximum
Age (years)	57.45	12.06	18	78
BMI (kg/m^2^)	27.82	3.55	19.60	36.20
Blood volume (mL)	5015.94	880.01	3527	7066
Tendons torn (No)	1.61	0.64	1	3
Tear size SSP/ISP (mm)	18.33	6.79	7	34
Operative time (min)	118.18	37.33	56	181
Suture anchors implanted (No)	3.57	1.34	1	8
Increase in irrigation fluid pressure (No)	20.27	10.72	2	52
Inflow irrigation fluid (L)	31.68	13.34	9	66
Outflow irrigation fluid (L)	25.94	12.50	3	54
Irrigation fluid retention (L)	2.59	0.94	1	5.50
Irrigation fluid retention (L/h)	1.39	0.49	0.39	2.80
Irrigation fluid retention (mL/min)	23.34	8.26	6.57	46.67
Retention rate (%)	9.41	4.88	2.47	31.11
Irrigation fluid retention as % body weight	3.23	1.34	1.06	7.64
Crystalloids (L)	3.08	0.58	2	4.50
Crystalloids + irrigation fluid retention (L)	5.67	1.18	3.80	9.50
Crystalloids + irrigation fluid retention % body weight	7.06	1.95	4.59	12.68
Hb in washed irrigation fluid (mg/100 mL)	20.23	4.76	7.79	27.36
MAP intraoperative (mmHg)	79.26	7.46	63.61	100.60
MAP postoperative (mmHg)	91.70	7.78	73	107

Abbreviations: BMI = body mass index; No = number; SSP = supraspinatus tendon; ISP = infraspinatus tendon; Hb = hemoglobin; MAP = mean arterial pressure.

**Table 2:** Preoperative and second-day postoperative blood parameters.

Variable	Mean	Std. Dev.	Minimum	Maximum
Preoperative blood Hb (g/dL)	14.47	1.27	11.80	17.50
Preoperative blood Hct	0.43	0.03	0.36	0.52
Preoperative blood RBC (10^12^/L)	4.93	0.45	4.14	5.99
Preoperative blood WBC (10^9^/L)	7.20	1.66	4.20	11.20
Preoperative blood PLT (10^9^/L)	244.10	47.18	167	387
Postoperative blood Hb (g/dL)	12.41	1.46	9.20	15.50
Postoperative blood Hct	0.37	0.042	0.28	0.46
Postoperative blood RBC (10^12^/L)	4.17	0.48	3.16	5.27
Postoperative blood WBC (10^9^/L)	7.72	2.09	4	14
Postoperative blood PLT (10^9^/L)	204.39	51	121	372

Abbreviations: Hb = hemoglobin; Hct = hematocrit; RBC = red blood cell; WBC = white blood cell; PLT = platelet.

**Table 3:** Hb drop and blood loss parameters.

Variable	Mean	Std. Dev.	Minimum	Maximum
Intraoperative Hb drop (g/dL)	0.11	0.06	0.01	0.29
Intraoperative blood loss (mL)	36.46	20.34	4.80	84.15
Intraoperative blood loss (mL/min)	0.29	0.11	0.08	0.57
Intraoperative Hb drop (%)	5.99	4.51	0.61	27.60
Intraoperative blood loss (%)	5.36	4.22	0.53	26.58
Intraoperative blood loss as % of estimated blood volume	0.76	0.45	0.11	1.90
Postoperative Hb drop (g/dL)	1.95	0.73	0.36	3.40
Postoperative (hidden) blood loss (mL)	754.72	277.93	136.42	1273.5
Postoperative Hb drop (%)	94.01	4.51	72.40	99.40
Postoperative (hidden) blood loss (%)	94.64	4.22	73.42	99.50
Postoperative blood loss as % of estimated blood volume	15.58	6.52	2.55	29.80
Total blood loss (mL)	791.17	280.96	185.81	1308.30
Total Hb drop (g/dL)	2.06	0.74	0.50	3.50
Total blood loss as % of estimated blood volume	16.34	6.66	3.48	30.60

Abbreviations: Hb = hemoglobin.

## Data Availability

The datasets generated and analyzed during the current study are available from the corresponding author on reasonable request.

## Abbreviations

The following abbreviations are used in this manuscript:

AC	Acromioclavicular
BMI	Body mass index
EPI	Epinephrine
GH	Glenohumeral
Hb	Hemoglobin
Hct	Hematocrit
ISP	Infraspinatus tendon
LHBB	Long head of the biceps brachii tendon
MAP	Mean arterial pressure
MFX	Microfractures
PLT	Platelet
RBC	Red blood cell
RCT	Rotator cuff tear
SA-BSTR	Subacromial bursectomy and soft tissue release
SC-BSTR	Subcoracoid bursectomy and soft tissue release
SSC	Subscapularis tendon
SSP	Supraspinatus tendon
TXA	Tranexamic acid
WBC	White blood cell
